# Acceptance and Attitude of Parents Regarding COVID-19 Vaccine for Children: A Cross-Sectional Study

**DOI:** 10.7759/cureus.24518

**Published:** 2022-04-27

**Authors:** Remiya Mohan, Vandna Pandey, Ashok Kumar, P. Gangadevi, Akhil Dhanesh Goel, Joyce Joseph, Nancy Kurien

**Affiliations:** 1 College of Nursing, All India Institute of Medical Sciences, Jodhpur, Jodhpur, IND; 2 Community Medicine & Family Medicine, All India Institute of Medical Sciences, Jodhpur, Jodhpur, IND; 3 Pediatric Nursing, All India Institute of Medical Sciences, Jodhpur, Jodhpur, IND

**Keywords:** attitude, acceptance, parents, vaccine, pandemic, covid-19

## Abstract

Background

The coronavirus disease 2019 (COVID-19) pandemic has claimed millions of lives worldwide. India also launched a COVID-19 vaccination drive, and clinical trials for a pediatric COVID-19 vaccine are in development.

Objectives

The study aims to assess the acceptance and attitude of parents regarding the COVID-19 vaccine for children in India. The study also aims to find the association between selected demographic variables and acceptance and attitudes in parents regarding the COVID-19 vaccine for children.

Materials and methods

We conducted a cross-sectional descriptive study with 204 participants, and data were collected online using Google Forms. The study participants were parents of children aged two to 15 years. A self-structured tool was used to assess parents' acceptance regarding vaccinating their children, and a modified vaccination attitudes examination scale was used to assess parents' attitudes toward pediatric vaccination against COVID-19. We used IBM SPSS Statistics for Windows, Version 20.0 (IBM Corp., Armonk, New York) to analyze the data. Demographic data were represented as frequency and percentage. Binary logistic regression analysis was used to determine the associations between sociodemographic data and parents' levels of acceptance and attitude. For all the data, p<0.05 was considered significant.

Results

The majority of the participants (85%) reported acceptance of the COVID-19 vaccine for children. More than 80% of parents agree that vaccines are essential to halt the COVID-19 pandemic and are mandatory for children. Most parents (62%) also believed that complementary medicine is better than vaccines for children. While most parents (95%) reported trusting the vaccine, but more than half (59%) reported concerns regarding the unknown future effects of the vaccine.

Mothers (odds ratio (OR), 2.963; p=0.015) and parents of children who received routine vaccination (OR, 0.175; p=0.039) were willing to vaccinate their children when a COVID-19 vaccine became available. Mothers (OR, 3.294; p=0.002) and respondents whose family member or close relative suffered from COVID-19 (OR, 0.420; p=0.029) accepted the COVID-19 vaccine irrespective of the child's age. Study participants who had previously tested positive for COVID-19 (OR, 0.275; p=0.012) believed vaccines for children were necessary to halt the COVID-19 pandemic.

Conclusion

We sought to assess parents' acceptance and attitudes regarding the COVID-19 vaccine for children in India. According to our results, while parents have a high acceptance of a pediatric COVID-19 vaccine, they also have a few apprehensions. Therefore, for a successful mass vaccination drive among the pediatric age group, there should be rigorous communication regarding the vaccine and staunch health campaigns to create more awareness and acceptance toward the COVID-19 vaccine for children.

## Introduction

The appearance of coronavirus disease 2019 (COVID-19) quickly became a pandemic. COVID-19 disrupted daily life in India and presented a serious challenge to physicians and scientists across the globe. Thankfully, children and adolescents seem to develop less severe diseases than adults. The World Health Organization (WHO) reported that global incidence among children under age five, children ages five to 14, and young people aged 15 to 24 is only 2%, 7%, and 15%, respectively [1]. Similarly, deaths worldwide in children up to age 14 have only occurred in 0.1% of cases and 0.4% of young people aged 15 to 24. Overall, only 0.5% of people aged 25 or younger have died due to COVID-19 [1]. However, there is a risk of long-term health effects for those who recover from COVID-19, and some patients develop multisystem inflammatory syndrome in children (MIS-C). MIS-C is a rare but serious condition that can manifest as anasarca [2].

India launched a vaccine against the virus responsible for COVID-19, the severe acute respiratory syndrome coronavirus 2 (SARS-CoV-2), on January 16, 2021 [3]. As of this writing, vaccination is not mandatory in India. COVID-19 vaccines for adults have already effectively gained acceptance in many countries. Clinical trials for COVID-19 vaccines in children as young as six months old are in progress [1]. Given the surge in COVID-19 cases and the emergence of the Omicron variant of concern, India began a vaccination drive with the Covaxin (Bharat Biotech, Hyderabad, India) vaccine in adolescents aged 15 to 18 years on January 3, 2022 [1,4,5]. The American Academy of Pediatrics recommends that children receive the COVID-19 vaccine once it is available [6]. Despite the effectiveness of vaccines for public health in general, global vaccination coverage has not improved recently, and some countries have reported a decline in vaccine coverage [7].

This study aimed to explore the acceptance and attitude of parents regarding the COVID-19 vaccine for children in India. The findings may help policymakers develop strategies that can reduce vaccine hesitancy prior to the launch of the pediatric COVID-19 vaccine in India.

## Materials and methods

Study design, setting, and sample

We conducted a cross-sectional descriptive study via online surveys of 204 parents of children aged two to 15 willing to participate and understand Hindi or English. The data were collected from July to September 2021. The surveys were converted to online forms using Google Forms, and a link was sent to subjects through email and WhatsApp. The completed forms that were received within the study window were coded and analyzed.

Study tools

Sociodemographic Proforma

We collected sociodemographic data from parents, including their age, gender (as father or mother), education, and occupation. We also collected data on past positive COVID-19 test results, the diagnosis of a family member with COVID-19, and any chronic illnesses or lifestyle-related diseases. We collected the age, gender, routine immunization status, and chronic illness or congenital disease history of the children.

Questionnaire for Vaccine Acceptance

A self-structured questionnaire consisting of eight items was used to assess the parental acceptance of the COVID-19 vaccine for their children. It used a five-point Likert scale, with responses ranging from strongly disagree (scored as a 1) to strongly agree (scored as a 5). The eight-item acceptance scale had high internal consistency, with a Cronbach's alpha of 0.806. Parents' responses were coded as "nonacceptance" of the COVID-19 vaccine for their children if marked as strongly disagree, disagree, and neutral, and the responses marked as agree and strongly agree were coded as "acceptance." The coding was reversed for the scoring of the question, "For children, naturopathy/Ayurveda is a better option than a vaccine."

Vaccination Attitudes Examination Scale

We used the standardized vaccination attitudes examination scale (VAX) to assess parental attitudes regarding the COVID-19 vaccine for their children [8]. This scale consists of 12 items under four parameters toward vaccine administration, including mistrust of vaccine benefits, worries about unforeseen future effects, concerns about commercial profiteering, and preference for natural immunity. The author was granted permission by the creator of VAX to modify the tool for use in this study. The tool was converted to use five-point Likert responses. Parents' responses were coded as "low negative attitude" for the COVID-19 vaccine for their children if they answered strongly disagree, disagree, and neutral, and responses marked as agree and strongly agree were coded as "high negative attitude." The coding was reversed for one item, the "mistrust of vaccine benefits" question.

Data collection procedure

The validated data collection tools were converted to Google Forms. The questions were prepared in Hindi and English. The link was created and circulated via email and WhatsApp. All respondents provided informed consent. The study design received ethical clearance from the Institutional Ethical Committee (AIIMS/IEC/2021/3540).

Data analysis

We used IBM SPSS Statistics for Windows, Version 20.0 (IBM Corp., Armonk, New York) to analyze the data. Demographic data were represented as frequency and percentage. Binary logistic regression analysis was used to find the association between sociodemographic variables and parental acceptance and attitude toward the vaccine. For all associations, p<0.05 was considered significant.

## Results

Demographic characteristics of participants

The study included responses from 204 participants, most of whom were younger than 40-years-old (83%). The mother-to-father ratio was approximately 1:1 (49.5% mothers, 50.5% fathers), and 50% of respondents had two children. Under half of the participants graduated from university (42.6%); nearly 43% were employed by the government, and just under half (48.5%) were nursing personnel. The majority of parents (87.7%) were vaccinated against COVID-19. Twenty-eight percent of respondents had tested positive for COVID-19. Most respondents (92.2%) had no chronic or lifestyle-related illnesses. Over half of respondents (68%) reported their children had received routine vaccinations, and 94.6% reported their children had no congenital or chronic diseases (Table 1).

**Table 1:** Demographic characteristics of participants (N=204). COVID-19: coronavirus disease 2019.

Demographic variable	Frequency (%)
Age (years)	
<40	169 (82.8)
>40	35 (17.2)
Relation with child	
Father	103 (50.5)
Mother	101 (49.5)
Number of children	
One	83 (40.7)
Two or more	121 (59.3)
Education	
Up to graduate	110 (53.9)
Postgraduate and above	94 (46.1)
Occupation	
Government/private job	152 (74.5)
Others	52 (25.5)
Type of occupation	
Health care provider	111 (54.4)
Non-health care provider	93 (45.6)
Vaccinated for COVID-19	
Yes	179 (87.7)
No	25 (12.3)
Ever been tested positive for COVID-19	
Yes	58 (28.4)
No	146 (71.6)
Family members/close relatives diagnosed with COVID-19	
Yes	89 (43.6)
No	115 (56.4)
Diagnosed with any chronic illness/lifestyle diseases	
Yes	16 (7.8)
No	188 (92.2)
Children vaccinated as per age	
Vaccinated	139 (68.1)
Not vaccinated	65 (31.9)
Child suffers from any chronic/congenital disease	
Yes	11 (5.4)
No	193 (94.6)

Acceptance of participants toward COVID-19 vaccine for children

**Figure 1:**
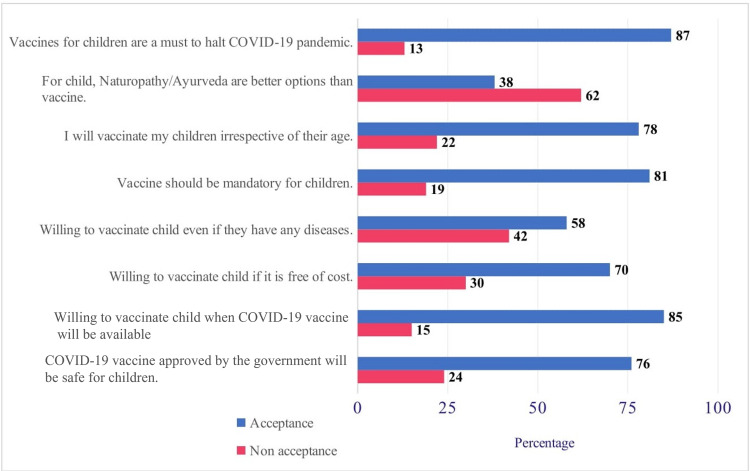
Acceptance of COVID-19 vaccine for children among parents. COVID-19: coronavirus disease 2019.

Item-wise acceptance and nonacceptance are presented in Figure 1. Most parents (85%) were willing to vaccinate their children, and 87% believed pediatric vaccines were necessary to halt this pandemic. More than 75% of parents believed that vaccines approved by the government would be safe and ready to vaccinate children irrespective of their age. Apart from the generally favorable acceptance, 62% of parents surveyed still believed complementary medicine is better than vaccines for children.

Table 2 depicts the association of parental acceptance of the COVID-19 vaccine for children with selected sociodemographic variables using binary logistic regression analysis. Acceptance was associated with mothers (odds ratio (OR), 2.963; p=0.015) and parents who vaccinated their children with routine vaccination (OR, 0.175; p=0.039). Mothers accepted the COVID-19 vaccine for their children even if their child had a chronic or congenital disease (OR, 1.961; p=0.028). Participants whose family member or close relative was diagnosed with COVID-19 considered the COVID-19 vaccine mandatory for children (OR, 0.352; p=0.014). Mothers (OR, 3.294; p=0.002) and participants whose family members or close relatives suffered from COVID-19 (OR, 0.420; p=0.029) accepted vaccination irrespective of the child's age. Respondents older than age 40 (OR, 3.541; p=0.023) and those with family members or close relatives who were not diagnosed with COVID-19 (OR, 2.235; p=0.025) regarded naturopathy (i.e., Ayurveda) as a better option than vaccines for children. However, respondents who tested positive for COVID-19 (OR, 0.275; p=0.012) considered vaccines for children essential to halt the COVID-19 pandemic.

**Table 2:** Association between acceptance and demographic characteristics of participants regarding the COVID-19 vaccine in children. *p-value is significant at <0.05. COVID-19: coronavirus disease 2019; OR: odds ratio.

Demographic characteristics	Vaccines approved by the government for COVID-19 will be safe for children. OR (p-value)	I will vaccinate my child when the COVID-19 vaccine will be available. OR (p-value)	I will vaccinate my child if it is free of cost. OR (p-value)	I will vaccinate my children even if they have any chronic or congenital diseases. OR (p-value)	Vaccines should be mandatory for children. OR (p-value)	I will vaccinate my children irrespective of age. OR (p-value)	For children, naturopathy/Ayurveda are better options as compared to vaccines. OR (p-value)	Vaccines for children are a must to halt the COVID-19 pandemic. OR (p-value)
Age (older or younger than 40 years)	0.580 (0.314)	0.606 (0.438)	1.074 (0.872)	1.854 (0.140)	0.617 (0.417)	1.420 (0.481)	3.541 (0.023)*	0.000 (0.998)
Relation with child (father or mother)	1.968 (0.057)	2.963 (0.015)*	1.729 (0.093)	1.961 (0.028)*	2.050 (0.075)	3.294 (0.002)*	0.964 (0.914)	1.831 (0.206)
Number of children (one vs two or more)	0.829 (0.593)	0.857 (0.720)	1.061 (0.855)	0.809 (0.496)	0.746 (0.457)	0.976 (0.949)	0.976 (0.944)	1.382 (0.494)
Education (graduate vs postgraduate or higher)	0.729 (0.375)	0.659 (0.345)	0.955 (0.887)	0.994 (0.984)	0.607 (0.223)	0.851 (0.673)	0.675 (0.251)	0.619 (0.317)
Occupation (government vs private job)	0.690 (0.414)	0.670 (0.464)	1.501 (0.300)	1.428 (0.350)	0.728 (0.521)	0.944 (0.900)	2.210 (0.093)	1.137 (0.826)
Type of occupation health care provider vs non-health care provider	0.975 (0.948)	1.357 (0.525)	0.938 (0.859)	0.697 (0.288)	1.163 (0.732)	1.013 (0.976)	0.788 (0.532)	0.959 (0.936)
COVID-19 vaccination status	0.923 (0.884)	0.527 (0.392)	1.181 (0.729)	0.984 (0.972)	2.586 (0.076)	1.772 (0.276)	4.123 (0.052)	2.312 (0.175)
Previously tested positive for COVID-19	0.543 (0.118)	0.980 (0.967)	0.643 (0.228)	1.130 (0.731)	0.559 (0.177)	0.494 (0.088)	0.924 (0.837)	0.275 (0.012)*
Family members/close relative diagnosed with COVID-19	0.895 (0.765)	0.501 (0.129)	0.877 (0.699)	1.273 (0.453)	0.352 (0.014)*	0.420 (0.029)*	2.235 (0.025)*	0.760 (0.574)
Diagnosed with any chronic/lifestyle diseases	0.703 (0.585)	0.571 (0.451)	1.014 (0.981)	0.556 (0.287)	0.561 (0.414)	0.584 (0.410)	0.807 (0.760)	0.407 (0.270)
Children up-to-date with routine vaccinations	0.711 (0.700)	0.175 (0.039)*	0.669 (0.616)	2.202 (0.379)	0.599 (0.588)	0.361 (0.247)	3.934 (0.119)	1.267 (0.849)
Child is suffering from chronic or congenital disease	0.394 (0.493)	1.261 (0.999)	2.051 (0.620)	1.082 (0.958)	1.481 (0.801)	2.812 (0.518)	5.442 (0.250)	0.194 (0.365)

Attitude of participants toward COVID-19 vaccine for children

**Figure 2:**
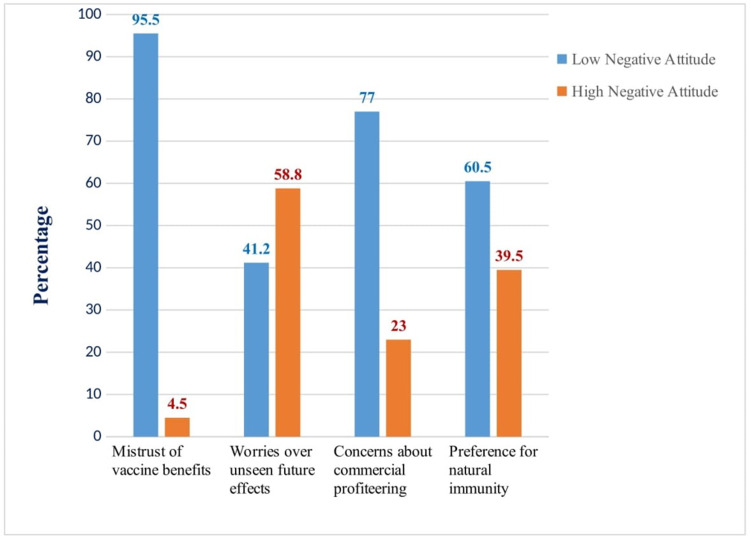
Attitude of parents regarding COVID-19 vaccine for children. COVID-19: coronavirus disease 2019.

Domain-wise attitude is presented in Figure 2, where "low negative attitude" is expressed as a positive attitude and "high negative attitude" is expressed as a negative attitude by participants toward the COVID-19 vaccine for their children. Most participants (95%) trusted the vaccine, but more than half (59%) had concerns regarding future, undiscovered adverse effects from the vaccine.

Table 3 depicts the association of parental attitudes toward the COVID-19 vaccine for their children using binary logistic regression analysis. Mothers (OR, 2.595; p=0.018) felt that children would be safe after vaccination. Mothers (OR, 3.191; p=0.015), participants who had not been vaccinated for COVID-19 (OR, 4.377; p=0.011), and those who had tested positive for COVID-19 (OR, 0.251; p=0.007) reported they rely on vaccines to stop COVID-19. Mothers (OR, 4.916; p=0.001) felt their children would be protected after getting vaccinated. Participants who graduated from college or university (OR, 0.445; p=0.035) and those diagnosed with chronic illnesses or lifestyle-related diseases (OR, 0.081; p<0.001) believed that although the vaccine may appear to be safe, there may be adverse effects that are not yet discovered. Participants older than age 40 expressed worry about future unknown effects of vaccines (OR, 3.132; p=0.008) and believed vaccines make large amounts of money for pharmaceutical companies but do not do much for regular people (OR, 3.009; p=0.017). The participants who never tested positive for COVID-19 (OR, 8.036; p=0.002) and those whose children have not received routine vaccinations (OR, 11.174; p=0.028) felt that vaccination programs are not useful. Participants older than 40 years (OR, 3.593; p=0.008), those not working in a government or private job (OR, 2.478; p=0.027), and those with family members or close relatives who have not been diagnosed with COVID-19 (OR, 2.173; p=0.023) believed natural immunity lasts longer than a vaccination. Participants who graduated from college or university (OR, 0.527; p=0.046) believed that natural exposure to viruses and germs gives better protection than vaccination. Participants who were not COVID-19 positive (OR, 2.553; p=0.010) reported that being exposed to diseases naturally is safer for the immune system than being exposed through vaccination.

**Table 3:** Association between attitude and demographic characteristics of participants regarding the administration of the COVID-19 vaccine in children. *p-value is significant at <0.05. COVID-19: coronavirus disease 2019; OR: odds ratio.

Demographic characteristics	I feel children will be safe after being vaccinated. OR (p-value)	I can rely on vaccines to stop COVID-19 infection. OR (p-value)	I feel my child will be protected after getting vaccinated. OR (p-value)	Although vaccine appears to be safe, there may be problems that we have not yet discovered. OR (p-value)	Vaccine can cause unforeseen problems in children. OR (p-value)	I worry about the unknown effects of vaccines in the future. OR (p-value)	Vaccines make a lot of money for pharmaceutical companies, but do not do much for regular people. OR (p-value)	Authorities promote vaccination for financial gain, not for people’s health. OR (p-value)	Vaccination programs are not useful. OR (p-value)	Natural immunity lasts longer than a vaccination. OR (p-value)	Natural exposure to viruses and germs gives the safest protection OR (p-value)	Being exposed to diseases naturally is safer for the immune system than being exposed through vaccination. OR (p-value)
Age (older or younger than 40-years-old)	0.639 (0.444)	0.228 (0.078)	0.257 (0.106)	0.447 (0.155)	2.390 (0.054)	3.132 (0.008)*	3.009 (0.017)*	3.031 (0.110)	4.557 (0.997)	3.593 (0.008)*	2.195 (0.105)	1.322 (0.561)
Relation with child (father or mother)	2.595 (0.018)*	3.191 (0.015)*	4.916 (0.001)*	0.992 (0.983)	0.828 (0.537)	0.752 (0.364)	1.260 (0.453)	0.696 (0.377)	0.329 (0.075)	1.126 (0.715)	0.674 (0.220)	1.199 (0.578)
Number of children (one vs two or more)	1.438 (0.363)	0.726 (0.479)	1.093 (0.842)	0.901 (0.778)	1.188 (0.574)	1.190 (0.581)	0.601 (0.104)	0.862 (0.718)	0.976 (0.966)	1.263 (0.470)	0.987 (0.967)	0.662 (0.215)
Number of children (one vs two or more)	0.778 (0.529)	0.475 (0.114)	0.446 (0.083)	0.445 (0.035)*	0.830 (0.543)	0.656 (0.183)	0.777 (0.414)	0.756 (0.493)	0.695 (0.535)	0.631 (0.158)	0.527 (0.046)*	1.319 (0.403)
Occupation (government vs private job)	0.735 (0.529)	0.405 (0.126)	0.386 (0.103)	0.602 (0.266)	0.650 (0.260)	0.778 (0.519)	1.251 (0.562)	0.709 (0.502)	1.003 (0.996)	2.478 (0.027)*	1.634 (0.239)	1.091 (0.834)
Type of occupation health care provider vs non-health care provider	1.038 (0.932)	1.768 (0.259)	1.199 (0.715)	1.749 (0.161)	0.797 (0.498)	1.305 (0.427)	1.016 (0.963)	0.818 (0.662)	640 (0.509)	1.522 (0.226)	1.380 (0.359)	1.348 (0.408)
COVID-19 vaccination status	1.093 (0.880)	4.377 (0.011)*	1.594 (0.462)	1.733 (0.298)	2.618 (0.059)	1.295 (0.587)	1.070 (0.890)	6.995 (0.074)	1.520 (0.668)	0.906 (0.849)	1.489 (0.454)	0.992 (0.988)
Previously tested positive for COVID-19	0.653 (0.323)	0.251 (0.007)*	0.403 (0.064)	2.109 (0.114)	1.596 (0.180)	1.670 (0.161)	1.899 (0.067)	1.562 (0.323)	8.036 (0.002)*	1.490 (0.281)	1.754 (0.118)	2.553 (0.010)*
Family members/close relative diagnosed with COVID-19	0.567 (0.167)	0.856 (0.747)	0.715 (0.473)	1.069 (0.867)	0.871 (0.668)	1.156 (0.660)	1.119 (0.726)	1.040 (0.927)	0.296 (0.069)	2.173 (0.023)*	0.962 (0.908)	0.906 (0.776)
Diagnosed with any chronic/lifestyle diseases	1.498 (0.624)	0.268 (0.080)	0.975 (0.977)	0.081 (0.001)	0.725 (0.584)	0.894 (0.843)	0.630 (0.435)	1.531 (0.551)	0.000 (0.998)	0.416 (0.180)	0.608 (0.437)	0.693 (0.560)
Children up-to-date with routine vaccinations	1.367 (0.784)	1.757 (0.657)	1.049 (0.967)	1.232 (0.841)	0.985 (0.986)	1.022 (0.978)	1.105 (0.903)	2.467 (0.335)	11.174 (0.028)*	1.572 (0.616)	3.863 (0.110)	3.379 (0.146)
Child is suffering from chronic or congenital disease	0.400 (0.580)	0.522 (0.728)	0.485 (0.679)	0.346 (0.485)	1.935 (0.619)	2.807 (0.466)	1.736 (0.678)	0.000 (0.999)	1.009 (0.996)	3.199 (0.441)	2.298 (0.563)	2.560 (0.513)

## Discussion

This study explored the acceptance and attitude of parents regarding the COVID-19 vaccine for children in India. While we identified several associations regarding vaccine acceptance, we also identified sociodemographic characteristics tied to vaccine hesitancy or low acceptance toward the COVID-19 vaccine for children. Policymakers might consider these findings while developing strategies to reduce vaccine hesitancy prior to the launch of the pediatric COVID-19 vaccine in India.

We found that 85% of parents are willing to vaccinate their children once the COVID-19 vaccine is available. This supports the report by Zhang et al. [9] wherein approximately 73% of parents accepted the COVID-19 vaccine for their children. In general, parents whose children received routine vaccinations were willing to have their children vaccinated against COVID-19. This association was also found by Altulaihi et al., who reported that parents with a high acceptance of the seasonal flu vaccine were also willing to accept the COVID-19 vaccine for their children [10]. We found that mothers were more likely to feel that children would be safe after vaccination and were willing to vaccinate children against COVID-19 than fathers. This positive attitude among mothers was also evident in Babicki et al.'s report, where mothers had a more positive attitude toward vaccination in children than fathers [11]. In addition, we noted that parents of children who were routinely vaccinated showed a willingness to vaccinate their children against COVID-19. Altulaihi et al. and El-Elimat et al. reported similar associations: those who took the seasonal influenza vaccine were more likely to accept COVID-19 vaccines [10,12].

One previous study found that healthcare professionals expressed a high level of willingness to have their children vaccinated against COVID-19 [13]. However, our results contrasted with this finding-we found that working in health care was not remarkably associated with vaccine acceptance. An unwillingness to vaccinate their children was a position shared by parents in three other studies, too: Yılmaz et al. reported that only 36.3% of their study population were willing to vaccinate [13], Babicki et al. reported a 44% willingness [11], and Altulaihi et al. reported that 53.7% were willing to vaccinate [10].

We identified several notable associations between certain sociodemographic factors and vaccine hesitancy or low willingness to vaccinate children against COVID-19. Respondents who were not highly educated and had chronic or lifestyle-related diseases believed that vaccines might have future unknown adverse effects, contributing to vaccine hesitancy. Participants older than 40 years expressed concerns regarding the unknown adverse effects of vaccines in the future and believed vaccines were profitable for pharmaceutical companies but not for regular people. This association of age with vaccine hesitancy was also reported by El-Elimat et al., who found that participants older than 35-years-old were less likely to accept the COVID-19 vaccine [12].

Over half of our respondents shared concerns focused on future adverse events due to the vaccine (59%). These concerns have been found in other studies and are worth addressing by policymakers [14,15]. Paul et al. found the most significant determinants of unwillingness and uncertainty were mistrust of vaccine benefits and adverse effects that have not yet been discovered [14]. Temsah et al. reported that 69% of parents refused a vaccine due to sparse safety information and 60.5% due to fears of vaccine side effects [15].

Regarding their opinion of policy, most parents (81%) in our study believed vaccinations should be mandatory for children, but another study did not share this majority. Babickie et al. reported that only 40.4% of parents felt vaccination should be mandatory [11]. Regardless of these perceptions, the COVID-19 pandemic has impacted all aspects of life. COVID-19 represented an extensive burden on mortality and morbidity and disrupted the worldwide economy.

The government should facilitate easy and safe access to vaccines and launch a campaign to encourage vaccination. Praveen et al. suggested that focusing on public fears before launching a mass vaccination program against COVID-19 will aid the campaign's success [16]. Carefully planned vaccination strategies will help overcome the current vaccine shortage [17].

Our study had a few significant limitations. The sample size was small and ethnically homogeneous, which potentially limits the generalizability of our findings. Future studies should be conducted with larger samples and in various ethnic groups to understand the mass acceptance and attitude of parents toward the COVID-19 vaccine for children.

## Conclusions

This study explored the acceptance and attitude of parents regarding the COVID-19 vaccine for children in India. While most parents were willing to vaccinate their children against COVID-19, we identified several characteristics of parents who were not willing to vaccinate. An unwillingness to vaccinate children against COVID-19 was common among parents who either themselves or loved ones had not been diagnosed with COVID-19, did not accept routine vaccinations, and had a strong belief in naturopathy/Ayurveda. Also, respondents older than 40 and those who were not highly educated and had chronic or lifestyle-related diseases were less willing to vaccinate their children against COVID-19. A common concern among those unwilling to vaccinate their children against COVID-19 was a fear of future unknown adverse effects from the vaccine. Policymakers should consider these findings while developing strategies to reduce vaccine hesitancy prior to the launch of the pediatric COVID-19 vaccine in India.

## References

[1] (2022). World Health Organization, interim statement on COVID-19 vaccination for children and adolescents. https://www.who.int/news/item/24-11-2021-interim-statement-on-covid-19-vaccination-for-children-and-adolescents..

[2] Made for Minds (2022). Made for minds. Risks and benefits of vaccinating children against COVID. https://www.dw.com/en/risks-and-benefits-of-vaccinating-children-against-covid/a-57507384.

[3] Jain J, Saurabh S, Kumar P (2021). COVID-19 vaccine hesitancy among medical students in India. Epidemiol Infect.

[4] (2022). Covaxin for children: facts you need to know. The Indian Express. https://indianexpress.com/article/parenting/health-fitness/covaxin-for-children-facts-health-immunity-parenting-7723293/.

[5] (2022). Ministry of Health and Family Welfare. Guidelines for COVID-19 vaccination of children between 15-18 years and precaution dose to HCWs, FLWs & 60+ population with comorbidities. https://www.mohfw.gov.in/pdf/GuidelinesforCOVID19VaccinationofChildrenbetween15to18yearsandPrecautionDosetoHCWsFLWs.

[6] (2022). COVID-19 vaccine: what parents need to know. Johns Hopkins Medicine. https://www.hopkinsmedicine.org/health/conditions-and-diseases/coronavirus/covid19-vaccine-what-parents-need-to-know.

[7] (2022). Vaccines and Immunizations. World Health Organization. https://www.who.int/health-topics/vaccines-and-immunization.

[8] Martin LR, Petrie KJ (2017). Understanding the dimensions of anti-vaccination attitudes: the vaccination attitudes examination (VAX) scale. Ann Behav Med.

[9] Zhang KC, Fang Y, Cao H (2020). Parental acceptability of COVID-19 vaccination for children under the age of 18 years: cross-sectional online survey. JMIR Pediatr Parent.

[10] Altulaihi BA, Alaboodi T, Alharbi KG, Alajmi MS, Alkanhal H, Alshehri A (2021). Perception of parents towards COVID-19 vaccine for children in Saudi population. Cureus.

[11] Babicki M, Pokorna-Kałwak D, Doniec Z, Mastalerz-Migas A (2021). Attitudes of parents with regard to vaccination of children against COVID-19 in Poland. A nationwide online survey. Vaccines.

[12] El-Elimat T, AbuAlSamen MM, Almomani BA, Al-Sawalha NA, Alali FQ (2021). Acceptance and attitudes toward COVID-19 vaccines: a cross-sectional study from Jordan. PLoS One.

[13] Yılmaz M, Sahin MK (2021). Parents' willingness and attitudes concerning the COVID-19 vaccine: a cross-sectional study. Int J Clin Pract.

[14] Paul E, Steptoe A, Fancourt D (2021). Attitudes towards vaccines and intention to vaccinate against COVID-19: implications for public health communications. Lancet Reg Health Eur.

[15] Temsah MH, Alhuzaimi AN, Aljamaan F (2021). Parental attitudes and hesitancy about COVID-19 vs. routine childhood vaccinations: a national survey. Front Public Health.

[16] Praveen SV, Ittamalla R, Deepak G (2021). Analyzing the attitude of Indian citizens towards COVID-19 vaccine-a text analytics study. Diabetes Metab Syndr.

[17] Lazarus JV, Ratzan SC, Palayew A (2021). A global survey of potential acceptance of a COVID-19 vaccine. Nat Med.

